# Empathy Among Internal Medicine Residents in a Community-Based Training Program: A Pilot Study

**DOI:** 10.15694/mep.2017.000077

**Published:** 2017-04-26

**Authors:** Halina Kusz, Jami Foreback, Anne Dohrenwend

**Affiliations:** 1 McLaren-Flint/Michigan State University

**Keywords:** Empathy, Post-graduate Training, Internal Medicine Residency

## Abstract

This article was migrated. The article was marked as recommended.

**Background:** Empathy in patient care is a highly valuable skill that is promoted in medical education; however, research conducted in academic centers strongly suggests a declining trend in empathy as years of medical education increase.

**Objective:** To assess residents’ empathy levels in a community-based internal medicine training program. We hypothesized that empathy in our program did not decrease with years of training.

**Methods:** A cross-sectional, observational study of 22 resident physicians who completed the Jefferson Scale of Physician Empathy between May and October of 2013. The residents were at the end of their first (PGY1), second (PGY2), or third (PGY3) year of training, or were incoming interns (PGY0) at the beginning of their first year.

**Results:** Of 48 eligible residents, responses of 22 (45%) are included in the analysis. The empathy scores for participants ranged from 96 to 136 with a mean of 117.4 and a SD of 10.1. Incoming residents, PGY1, 2 and 3 residents’ mean scores were 109.7, 117.2, 114.3 and 124.0 respectively. There was no statistical difference between males and females or between PGY1 and PGY2 residents. A statistically significant difference in mean empathy scores was found between incoming residents and PGY3, with PGY3 residents scoring higher in empathy.

**Conclusion:** The empathy scores in our internal medicine residency program identified higher levels of empathy associated with residents at the end of training. This may be related to our targeted curricula which includes behavioral science and geriatric medicine curriculums.

## Background

There are many valuable skills in the medical profession, but the ability to be empathic is perhaps one of the most desired. While there is no universally accepted definition of empathy, there are points of general agreement. In medical education and patient care settings, empathy is usually defined as a cognitive ability to understand and respond to the patients’ thoughts, feelings and concerns ( Hojat, Gonnella, Nasca, Mangione, Vergare et al., 2002; Halpern, 2003). Empathy confers many benefits to patients and physicians alike. Empathy has been associated with increased patient satisfaction, decreased patient anxiety, increased adherence, and better clinical outcomes ( Hojat et al., 2011; Del Canale et al., 2012; Derksen et al., 2013). Physicians who score high on measures of empathy demonstrate lower levels of burnout and a higher sense of professional accomplishment ( Shanafelt et al., 2005; Lamothe et al., 2014; Thirioux et al., 2016). Empathetic physician communication reduces major medical errors, and is associated with fewer malpractice lawsuits ( Beckman et al., 1994; Levinson et al., 1997; Stelfox et al., 2005). Despite the benefits that empathy offers to patients and physicians, and despite the strong support empathic skill development has received from the medical education community, studies show that empathy among undergraduates and postgraduates declines with years of training ( Hojat et al., 2009; Neumann et al., 2011). The most significant decline occurs during the 3rd ( Hojat et al., 2009) year of training with continued decline across three years of internal medicine residency training ( Bellini et al., 2002; Mangione et al., 2002; Bellini et al., 2005). A few studies counter this assertion. In studies of interventions designed to increase empathy, some interventions resulted in higher empathy scores ( Hojat et al., 2005; Hojat et al., 2013). In response to the observed trends in medical trainees’ empathy, many new methods are being developed to preserve and promote empathy ( Hojat et al., 2013; Batt-Rawden et al., 2013; Kelm et al., 2014). Some of the ways empathy is taught in the medical profession include reflective writing ( Shapiro et al., 2006), teaching through drama or role-playing ( Lim et al., 2011), communication skills training ( Shield et al., 2011), and brief courses ( Riess et al., 2012). The declining trend in empathy in medical students and residents should be cause for concern. Are physicians losing the ability or desire to practice empathy-based medicine? Are the declining skills of empathetic care overestimated, and is there need for further study? ( Colliver et al., 2010; Roff, 2015). The primary objective of our study was to assess empathy levels among our internal medicine residents by level of training. Based on our observations of residents’ clinical care, we hypothesize that our residents would not show declining levels of empathy with increased years of training, and this preservation and/or development of empathic skills might be attributed to our targeted curricula.

## Methods

**Participants:** Twenty two internal medicine resident physicians at McLaren-Flint hospital, a community-based training program affiliated with Michigan State University. The three year program consists of 12 residents in each post-graduate year (PGY) 1, 2, and 3. These residents were surveyed at the end of the academic year (May-June 2013). In addition, we included 12 in-coming residents. The incoming residents were surveyed at the beginning of their intern year (July-October 2013). More than 95% of the residents are international graduates.

**Instrument:** To assess empathy levels among our residents, we used the well validated and widely used Jefferson Scale of Empathy (JSPE) Health Professional-version for physicians and practicing health professionals. The survey is self-administered. It includes a 20 item test that is answered by using a 7 point Likert-type scale (1 = strongly disagree and 7 = strongly agree). ( Hojat, Gonnella, Nasca, Mangione, Veloksi et al., 2002).

**Procedures:** The study received an exemption from our Institutional Review Board. Consent for the study was implied by completion of the voluntary survey, as described in a brief cover letter. Participation in the study was confidential and voluntary. Participant identifier codes (ID) were assigned to each participant by support staff with no relation to the study. Participants received their code via e-mail and were asked to sign in to the on-line survey site using their assigned IDs. To increase our response rate we sent two email reminders. The survey was administered on line from May to October 2013 via the Thomas Jefferson University website. In addition to the empathy survey, residents were asked to complete a demographic questionnaire that included three questions: age, gender, and level of training.

**Statistical Analysis:** The calculation of empathy scores and statistical analysis were performed by Jefferson University. The JSPE scoring report included: descriptive statistics (mean, standard deviation, range, mode and quartiles), a histogram with distribution of score for the group, and empathy scores for each resident as identified by their confidential participant code. An additional report included gender and group comparisons. A t-test was used to determine if statistically significant differences were evident between males and females. Analysis of Variance (ANOVA) was used to compare empathy scores by post-graduate year.

## Results

The total number of resident physicians surveyed was forty eight. Twenty seven residents (53%) responded to the survey. Among them, 5 responders were not able to be identified by the level of training and their data was excluded. Therefore, data obtained from 22 responders (45% response rate) was analyzed. When comparing empathy scores by gender, there are 21 observations and 1 missing. Our sample includes 14 men (67%) and 7 women (33%), ages 21-30 (71%) and 31-40 (28%). (See Table 1)

**Table 1.** Demographic Data of Participants

AGE	Number	Percent
21-30	15	71
31-40	6	29

GENDER	Number	Percent
Female	7	33
Male	14	66

Individual empathy scores on the JSPE range from 20 to 140 with higher scores indicating higher empathy levels. In studies of “people oriented” specialty physicians, the mean empathy score is 120 with a range of 50 to 140 and standard deviation (SD) of 12 ( Hojat, Gonnella, Nasca, Mangione, Veloksi et al., 2002). The empathy scores among our participants ranged between 96-136 with a cumulative mean score of 117.4 and a SD of 10.1. The empathy score distribution for the sample showed a bell-shaped pattern.

The mean empathy score for female participants was 119.9 with a SD of 7.7. The mean empathy score for men was 115.6 with a SD of 11.2. A t-test comparing empathy scores for men and women was non-significant, possibly due to our small sample size (t _(19)_ = 1.03; p = 0.32). The mean scores for incoming residents and PGY1-3 were 109.7, 117.2, 114.3 and 124.0 with SDs of 6.8, 8.5, 12.3, and 7.2 respectively. (See Table 2)

**Table 2.** Participants JSPE score compared by post-graduate year of training

Year of Training	N	Mean Score	Standard Deviation
In-coming *	3	109.7	6.8
PGY-1	5	117.2	8.5
PGY-2	7	114.3	12.3
PGY-3 *	7	124.0	7.2

* Statistically significant in Analysis of Variance (ANOVA)

Abbreviations: JSPE, Jefferson Scale of Physician Empathy; PGY, post-graduate year.

**Figure 1.**
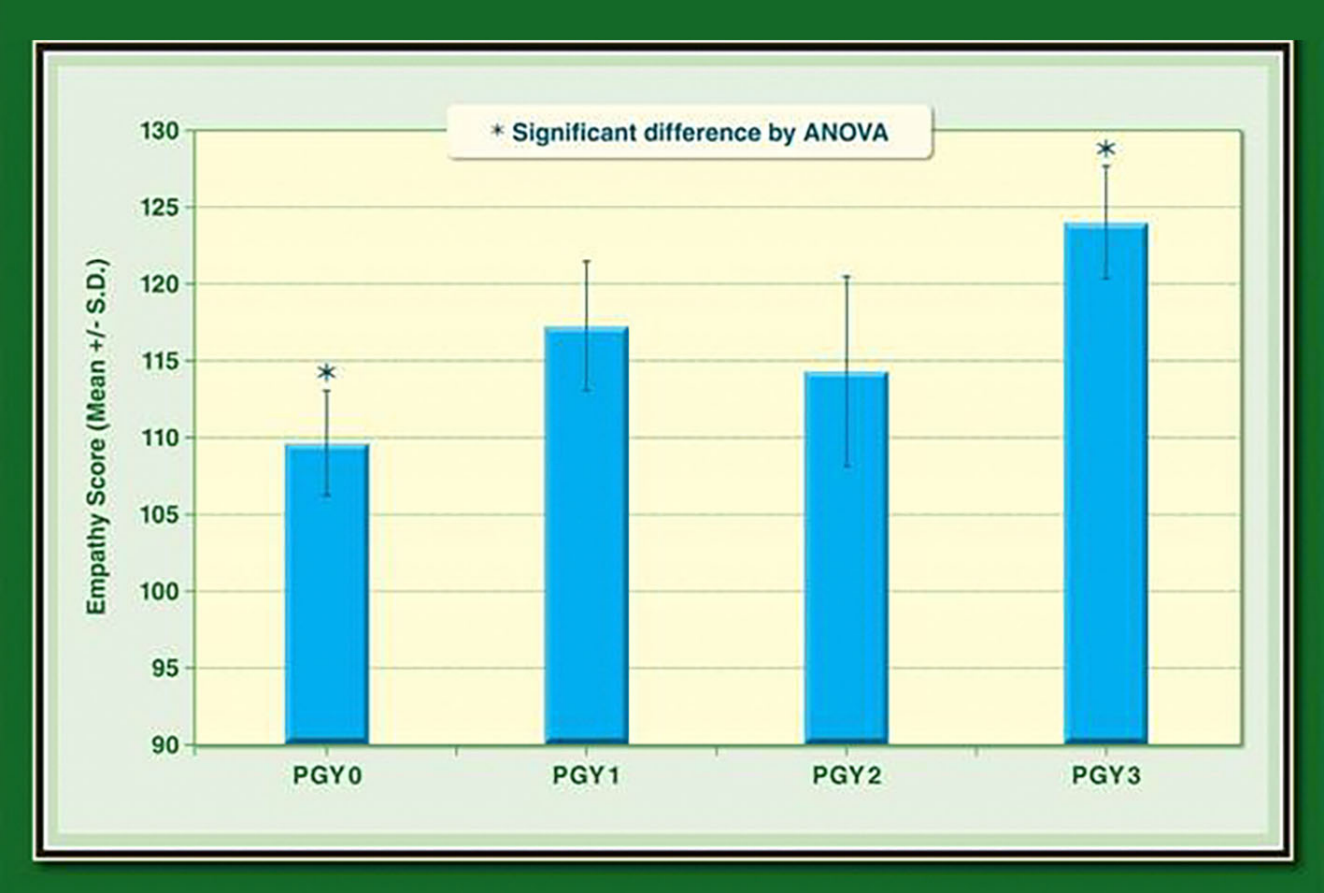
Residents' Empathy Scores by PGY.

A statistically significant difference in mean empathy scores was found between incoming and PGY3 residents (See Figure 1). There were no statistically significant differences between mean empathy scores for other groups.

## Discussion

The goal of our study was to assess the level of empathy in our residents by level of training and compare the results to those found in the literature.

To our knowledge, this is the first study of empathy in a community-based residency program. In the U.S., most internal medicine training programs are community-based and are comprised predominantly of international medical graduates making this cohort an important one to consider in discussions regarding whether empathy decreases as the years of training increase.

Our internal medicine residents cumulative, mean empathy score was 117.4 with a SD of 10, which is comparable to U.S. practicing physicians’ scores in “people-oriented” specialties.

For “people-oriented specialties,” defined as family medicine, internal medicine, pediatrics and psychiatry, the mean empathy score was 121.7 with a SD of 10.6 ( Hojat, Gonnella, Nasca, Mangione, Veloksi et al., 2002). These scores are similar to our residents’ scores despite our residents’ younger ages and status as “resident physicians.” Our residents’ ages range from 21 to 40 years, compared to physicians’ ages that range from 29 to 87 years. Our residents are trained in a small, community hospital, compared to practicing physicians’ who identify as being from large, urban, university systems. Our results suggest that our physicians’ empathy scores, as a group, are not different from much larger data sets in similar specialties.

Although PGY1 and PGY2 residents’ empathy scores appeared to be different when compared to PGY3 scores (See Figure 1), no significant statistical difference was found, possibly due to the small sample size. Despite the small sample size, there was a statistically significant difference between in-coming residents and PGY-3 residents.

**Figure 2.**
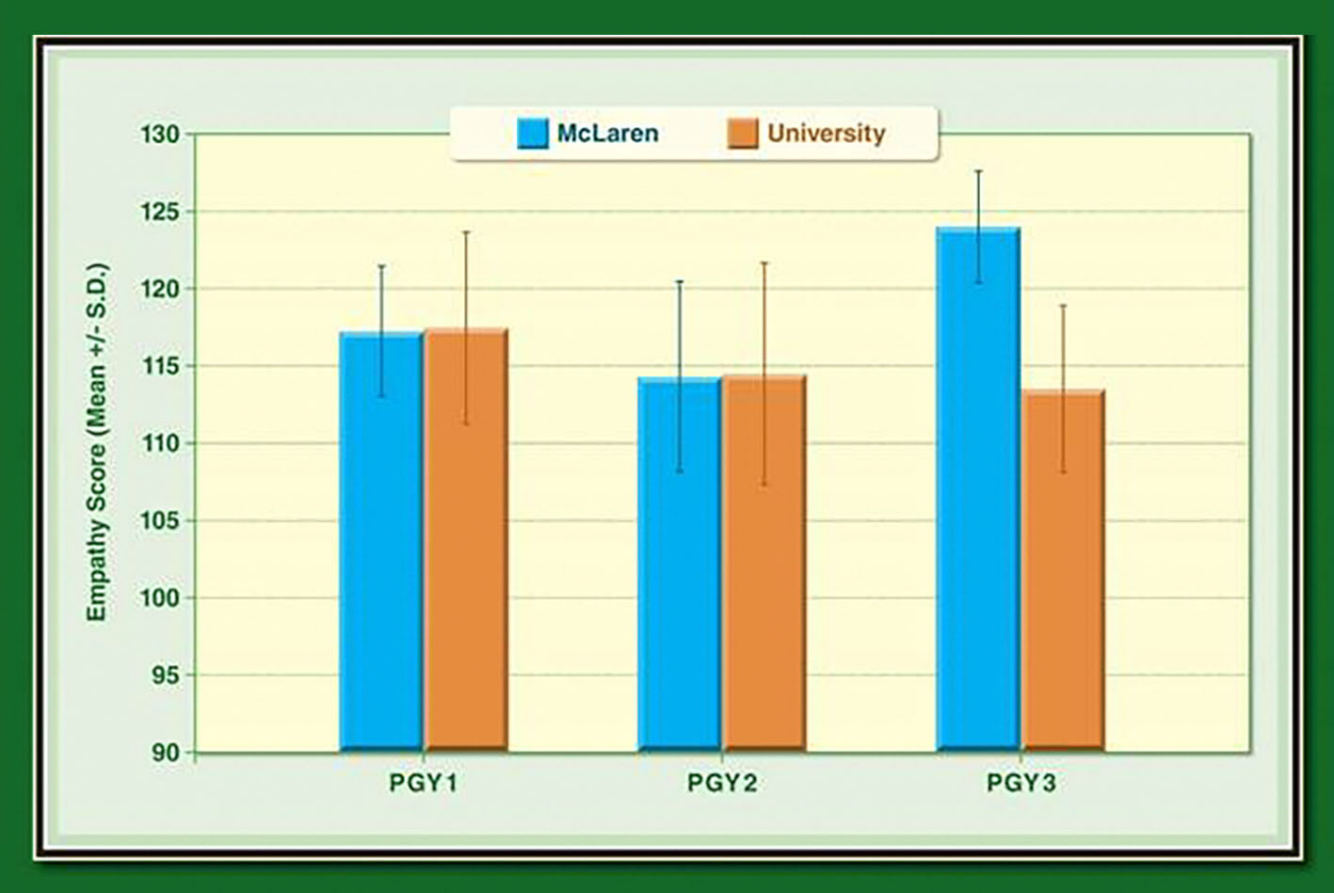
McLaren vs. University Internal Medicine Residents.

In the literature, mean empathy scores among internal medicine residents in university-based training program were 117.5, 114.5, and 113.5 with a SD of 12.4, 14.3 and 10.8 for PGY1, PGY-2 and PGY-3, respectively ( Mangione et al., 2002), (See Figure 2) As shown in Figure 2, our mean empathy scores for PGY1, PGY2 and PGY3 level were 117.2, 114.3, and 124.0 with SDs of 8.5, 12.3, and 7.2, respectively. This is very similar for PGY1 and PGY2 residents training in university settings. Considerably differences are found in mean empathy scores of PGY3 residents training in university-based systems compared with those training in community-based systems. This disparity may be explained by differences between programs, i.e. university vs. community-based, or between residents, due to the prevalence of international medical graduates in our sample.

Our finding of higher empathic levels in PGY3 residents compared with incoming residents runs counter to previous studies which show an inverse trend between empathy and years of training. This finding may be due to an intentional training effort. Our program has full-time behavioral medicine/psychologist and geriatric faculty and protected time for behavioral science and geriatric medicine curriculums. These curricula were developed and implemented 15 years ago with a shared curricular goal to teach empathetic and compassionate care of patients.

Our Behavioral Medicine curriculum includes a seven half-day block rotation in behavioral science for PGY1 residents and the following longitudinal elements supervised by our psychologist faculty: twice a month lectures, video-tape review of patient encounters, shadowing of patient encounters, and, as needed, clinic consultations.

Our Geriatric Medicine curriculum is supervised by a geriatrician and includes a one-month geriatric block rotation with a longitudinal nursing home experience assigned to PGY2 and PGY3 residents.

Skills learned during behavioral science rotation are practiced throughout residency training. However, empathy is especially reinforced on the geriatric rotation through direct observation of residents’ interactions with older and demented patients. There is a clear priority placed on communication skills by our program director and supported by our faculty.

**Study Strengths and Limitations:** This is a small study in a community-based teaching hospital. In a small sample, the study is “under powered” and significant differences might be missed. Another limitation is that we are unable to compare gender and age differences by PGY level. This is due to the confidential method by which data is provided from Jefferson University. This survey is self-reported rather than objectively measured. Our study is also from one institution and at a single point in time, which reduces generalizability. Next steps include a larger study including all three teaching hospitals in our community.

There are many possibilities for the finding of higher self-reported empathy in our third year residents compared with incoming residents. Empathy levels of individuals from each PGY level were not assessed prior to entering the program. The PGY3 group may simply have some particularly empathic individuals in the cohort and/or PGY0 group some less empathic individuals. An alternative study design might focus on within-subject empathy development over the course of residency. Utilizing a before and after design, each resident’s empathy scores would be assessed at program acceptance and yearly thereafter.

**Study Implications:** Currently, many different methods of teaching and preserving empathy in medical trainees are under evaluation. We suspect that our finding of higher levels of empathy among PGY3s as compared with incoming residents may be the result of prioritizing empathy training in our internal medicine program. Other informal aspects of resident training might contribute to resident empathy development, such as faculty role-modeling, stated and unstated norms and expectations, and how programs respond to residents’ emotional needs, thereby modeling empathy by responding empathically toward residents.

## Conclusions

We conclude that empathy may not decline during residency years. Providing trainees with specially designated empathy training may influence their clinical empathy development. More studies of empathy, including longitudinal studies, are needed to accurately reflect empathy trends in the context of targeted curricula and community-based programs.

## Take Home Messages

• Residents’ empathy in patient care may not decline.
• Specially designated training programs may influence empathy development.
• Integrated behavioral science and geriatric medicine curriculum offer substantial promise to fulfill this professional requirement.

## Notes On Contributors

Dr. Halina Kusz, MD, FACP, AGSF is an Associate Professor in the Department of Medicine, Michigan State University; a fellowship-trained educator with more than 15 years’ experience in leading a unique geriatric curriculum: a one-month block rotation combined with a longitudinal nursing home experience at McLaren-Flint’s internal medicine residency program.

Dr. Jami Foreback, MD, PhD, FACP, is an Associate Professor in the Department of Medicine, Michigan State University, fellowship-trained educator with 14 years of experience as internal medicine. She developed a strategic plan for research in the residency program and led the quality improvement program for internal medicine residents.

Dr. Anne Dohrenwend PhD, is an Associate Professor in the Department of Medicine, Michigan State University; fellowship-trained in Clinical Health Psychology; developed and leads the Behavioral Science curriculum at McLaren-Flint’s internal medicine residency program that includes: block rotation, didactic sessions, video-taping and teaching empathetic communication skills.
