# Supplementary material for: Empathy Among Internal Medicine Residents in a Community-Based Training Program: A Pilot Study
Source: MedEdPublish (2016). 2017 Apr 26;6:77. [Version 1] doi: 10.15694/mep.2017.000077 (PMC10885251; doi:10.15694/mep.2017.000077)
Supplement: Peer review history

# Reviewer report: Reviewer response for version 1

**DOI:** 10.21956/mep.19292.r28074

**Published:** 2018-06-08

**Authors:** Anthony David.M

**Affiliations:** 1 Soho

This review has been migrated. The reviewer awarded 3 stars out of 5 An interesting study. The need for empathy is increasing as the complexity of health care delivery systems is increasing these days. Some patients are dazzled and need empathetic physicians to guide them through the maze of medical technology.A longitudinal study of how the patients of these doctors rated them would be an excellent followup for this study.

Reviewer Expertise:

NA

No decision status is available

# Reviewer report: Reviewer response for version 1

**DOI:** 10.21956/mep.19292.r28078

**Published:** 2017-05-16

**Authors:** Susmita Reddy Karri

**Affiliations:** 1 Topiwala national medical college and BYL nair charitable hospital, mumbai. india

This review has been migrated. The reviewer awarded 4 stars out of 5 Well designed study. Can form the basis of larger studies in future.Empathy is an important factor in patient care. The paper shows that empathy can be 'taught.'However, some countries/ states have empathy or moral science in their primary school curriculum as well.. It would be interesting to see if students who 'learn' in primary school are more empathetic towards patients. I hope to see a larger study with more logitudinal interventions in future.

Reviewer Expertise:

NA

No decision status is available

# Reviewer report: Reviewer response for version 1

**DOI:** 10.21956/mep.19292.r28076

**Published:** 2017-04-28

**Authors:** Gert Van Zyl

**Affiliations:** 1 University of the Free State

This review has been migrated. The reviewer awarded 4 stars out of 5 What an important area and an excellent start to a process of continued research to provide us with answers on how to improve empathy as part of our curriculum. Looking forward to some more work on this topic!

Reviewer Expertise:

NA

No decision status is available

# Reviewer report: Reviewer response for version 1

**DOI:** 10.21956/mep.19292.r28073

**Published:** 2017-04-27

**Authors:** Subha Ramani

**Affiliations:** 1 Harvard Medical School, Brigham and Women's Hospital

This review has been migrated. The reviewer awarded 5 stars out of 5 Importance of the topic:Fostering empathic patient care is clearly an important topic and the authors have conducted a very nice pilot study and demonstrated at least no steep decline between PGY1-3 residents. Strengths of the paper: It is a well written paper, well referenced with sound methodology. Though a small study, it could be the launching point of more substantive research into this area. The study, bibliography, study design etc would be of value to educators in any healthcare discipline as empathy should be no different in medical vs allied disciplines. It is particularly essential in interprofessional curricula and teamwork in clinical settings.Limitations: As the authors state, it is a small study in a very specific setting. While I commend the program for some interventions, it may be challenging to attribute the non decline in empathy to 2 defined curricula offered at different points of the training. Future research: Studying larger numbers of participants in different disciplines, designing more longitudinal interventions to enhance empathy and systematically studying their impact, and comparing results across community vs urban settings would all add value.Last but not least, this is an area that begs a study design using qualitative methodology.

Reviewer Expertise:

NA

No decision status is available

# Reviewer report: Reviewer response for version 1

**DOI:** 10.21956/mep.19292.r28075

**Published:** 2017-04-26

**Authors:** Richard Hays

**Affiliations:** 1 James Cook University

This review has been migrated. The reviewer awarded 4 stars out of 5 It is important to document a situation where empathy increases during training and experience. The numbers are small and it is not clear to me what was done differently in the training program, but reports are very welcome that are different to the usual (where learners become somehow 'hardened' or more indifferent through at least more junior training). The next steps are to see if this can be replicated elsewhere and then to unpack the 'black box' of the training program to see if positive influences can be identified. It may be that the more comprehensive, longitudinal care model often used in residency training allows for a different kind of experience of patient journeys? Worth investigating!

Reviewer Expertise:

NA

No decision status is available

# Reviewer report: Reviewer response for version 1

**DOI:** 10.21956/mep.19292.r28077

**Published:** 2017-04-26

**Authors:** Trevor Gibbs

**Affiliations:** 1 AMEE

This review has been migrated. The reviewer awarded 4 stars out of 5 An interesting paper that touches upon a very important subject. Given that most faculty and curriculum designers would agree that empathy is a key characteristic in any healthcare practitioner and a key feature in curricula, it is disquieting to read the various papers that suggest the decline in empathy over the undergraduate years. Although this paper is based on a small group of students and reflects one particular course in one particular institution, it does demonstrate that empathy can be "taught" and perhaps " learned".We now need to investigate which methodology , approach etc. can promote the development of empathy

Reviewer Expertise:

NA

No decision status is available
